# Analysis of Genotype-Phenotype Correlations in Patients With Degenerative Dementia Through the Whole Exome Sequencing

**DOI:** 10.3389/fnagi.2021.745407

**Published:** 2021-10-14

**Authors:** Lin Sun, Jianye Zhang, Ning Su, Shaowei Zhang, Feng Yan, Xiang Lin, Jie Yu, Wei Li, Xia Li, Shifu Xiao

**Affiliations:** Alzheimer’s Disease and Related Disorders Center, Shanghai Mental Health Center, Department of Geriatric Psychiatry, Shanghai Jiao Tong University School of Medicine, Shanghai, China

**Keywords:** Alzheimer’s disease, frontotemporal lobe degeneration, dementia, next-generation sequencing, whole exome sequencing (WES)

## Abstract

**Background:** Sporadic dementias generally occur in older age and are highly polygenic, which indicates some patients transmitted in a poly-genes hereditary fashion.

**Objective:** Our study aimed to analyze the correlations of genetic features with clinical symptoms in patients with degenerative dementia.

**Methods:** We recruited a group of 84 dementia patients and conducted the whole exome sequencing (WES). The data were analyzed focusing on 153 dementia-related causing and susceptible genes.

**Results:** According to the American College of Medical Genetics and Genomics (ACMG) standards and guidelines, we identified four reported pathogenic variants, namely, *PSEN1* c.A344G, *APP* c.G2149A, *MAPT* c.G1165A, and *MAPT* c.G742A, one reported likely pathogenic variant, namely, *PSEN2* c.G100A, one novel pathogenic variants, *SQSTM1* c.C671A, and three novel likely pathogenic variants, namely, *ABCA7* c.C4690T, *ATP13A2* c.3135delC, and *NOS3* c.2897-2A > G. 21 variants with uncertain significance in *PSEN2*, *C9orf72*, *NOTCH3*, *ABCA7*, *ERBB4*, *GRN*, *MPO*, *SETX*, *SORL1*, *NEFH*, *ADCM10*, and *SORL1, etc.*, were also detected in patients with Alzheimer’s disease (AD) and frontotemporal dementia (FTD).

**Conclusion:** The new variants in dementia-related genes indicated heterogeneity in pathogenesis and phenotype of degenerative dementia. WES could serve as an efficient diagnostic tool for detecting intractable dementia.

## Introduction

Dementia currently affects an estimated 46.8 million people globally (Chaudhury et al., 2018). The most common dementia is Alzheimer’s disease (AD), and others are less common, such as dementia with Lewy body (DLB), frontotemporal dementia (FTD), and Huntington’s disease (HD), *etc*. The etiology of most neurodegenerative dementias is considered multifactorial, including genetic and environmental factors (Nicolas and Veltman, 2019). A proportion of dementias with a positive family history are consistent with a single gene pattern of inheritance, which provides an opportunity to make a precise diagnosis in the very early stages of disease (Koriath et al., 2020). However, sporadic dementias generally occur in older age and are highly polygenic, which indicates some patients transmitted in a poly-genes hereditary fashion.

It is known that dormant inherited mutations relating to amyloid β (Aβ) synthesis in familial AD include *APP*, *PSEN1*, and *PSEN2*, and variants relating to the deposition of multiple abnormal proteins in FTD include *MAPT*, *GRN*, *SQSTM1*, *TARDBP*, *C9orf72*, and *ERBB4, etc* (Sun et al., 2020). However, mutations in these genes can only explain 13% of early-onset AD (EOAD) and 60% of familial FTD, respectively (Bettens et al., 2013; Olszewska et al., 2016). At present, the *APOE* ε4 allele is the strongest genetic factor of sporadic AD, which confers a 3- to 15-fold increased risk of AD (Seo et al., 2020). Through Genome-Wide Association Studies (GWASs) and next-generation sequencing, some genetic loci have been discovered, which affect the risk of sporadic AD and other dementias, including the *TREM2*, *ABCA7*, *NOTCH3*, *TRIP4*, *ATP13A2, and ABI3* genes (Chaudhury et al., 2018; Nygaard et al., 2019), while the clinical phenotypes relating to these variants are not fully known.

Recently, whole exome sequencing (WES) has been demonstrated to be an efficient tool for detecting novel pathogenic or risk variants in large samples and delivering novel insights with these selected patients (Xu et al., 2018). In this study, considering the limitation of WES to detect copy number variations (CNVs), we have screened the *C9orf72* gene in patients suspected of FTD. In this study, we discuss the correlations between genotype and clinical phenotype of degenerative dementias, which increases interest in the search for novel variants as candidate causal mechanisms in some patients with a sporadic presentation through WES.

## Materials and Methods

### Subjects

A total of 84 patients were included in our study. All patients were assessed by specialists in the field of dementia. Of note, 55 patients fulfilled the diagnostic criteria for probable AD (McKhann et al., 2011), 14 patients met the clinical criteria for the FTD disease spectrum (Gorno-Tempini et al., 2011; Rascovsky et al., 2011), 6 patients fulfilled the diagnostic criteria for dementia but with uncertainty whether possible AD or FTD and nine patients could not be judged the dementia type or fulfilled other types of neurodegenerative dementia such as DLB. All available affected individuals were recruited from the Geriatric Psychiatry Department of Shanghai Mental Health Center. The research was approved by the Ethics Committee of Shanghai Mental Health Center. Written informed consents were obtained from all subjects.

### Examinations

All patients received neuropsychological assessments including Mini-Mental State Examination (MMSE) or Montreal Cognitive Assessment (MoCA). Brain imaging and polymorphisms of *APOE* were analyzed in all patients. Blood tests (i.e., treponema pallidum hemagglutination assay, HIV assay, vitamin B12 levels, folic acid levels, thyroid function, and tumor markers) were conducted to exclude acquired causes of dementia.

### Genetic Analysis

To comprehensively explore the potential genetic factors of the involved patients, we summarized 153 dementia-related causing and susceptible genes using PubMed database and Online Mendelian Inheritance in Man (OMIM) (Table 1). Total genomic DNA was prepared and amplified from the peripheral blood of all subjects according to the standard procedures. The quality of DNA was assessed by Qubit 3.0 (Thermo Fisher Scientific, United States) and agarose gel electrophoresis. WES was performed by HiSeq X Ten (Illumina, United States), and the sequencing library was carried out according to the SureSelectXT Target Enrichment System Manual (Agilent, United States). The average depth of coverage was 119.53. The data were analyzed for single nucleotide polymorphism (SNP) and insertion or deletion (INDEL) based on the genome analysis toolkit (GATK) best practice. The significant results were comprehensively evaluated in aspects, including minor allele frequency (MAF), conservation, predicted pathogenicity, disease association, confirmation with Sanger sequencing, and familial segregation. Alignment to the human genome assembly hg19 was carried out, followed by recalibration and variant calling. First, population allele frequencies compiled from public databases of normal human variation (dbSNP, National Center for Biotechnology Information Database of Single Nucleotide Polymorphisms 142; ESP6500, National Heart, Lung and Blood Institute Exome Sequencing Project 6500; gnomAD version 2.1.1, Genome Aggregation Database; ClinVar; HGMD, Human Gene Mutation Database; and 1000g, 1000 Genomes Project) were used to initially filter the data set to exclude all variants presenting in the population at greater than 5% frequency. A secondary filter for a paroxysmal movement disorder gene panel was then applied. A total of 200 normal Chinese individuals were set as control, and all the variants in this study could not be detected in the control group. The software of Mutation Taster, Plyphen2, and Mendelian Clinically Applicable Pathogenicity (M-CAP) Score were applied to predict the pathogenicity of the detected variants. These results were interpreted based on the American College of Medical Genetics and Genomics/Association for Molecular Pathology (ACMG/AMP) standards and guidelines (Li et al., 2017).

**TABLE 1:** Dementia-related causing and susceptible genes.

A2M	BSCL2	DNAJC5	GRIA2	MAP3K14	PLEKHG5	SLC5A7	TRPV4
ABCA7	C19orf12	DNMT1	GRID2	MAPT	PNPLA6	SMN1	TUBA4A
ABCD1	C9orf72	DPP6	GRIK1	MAPT	PRICKLE1	SMN2	TYROBP
ACE	CAMK1G	DYNC1H1	GRN	MATR3	PRKAR1B	SNCA	UBA1
ADAM10	CASP3	EFHD2	GSK3B	MEF2C	PRKN	SNCB	UBQLN2
ALS2	CD2AP	ELP3	HFE	MOB3B	PRNP	SOD1	UNC13A
ANG	CD33	EPHA1	HLA-DQB1	MPO	PRPH	SORL1	UNC5C
APBB2	CELF1	EPM2A	HNRNPA1	MS4A4E	PRPH2	SPAST	VAPB
APEX1	CHCHD10	ERBB4	HNRNPA2B1	MS4A6A	PSEN1	SPG11	VCP
APOE	CHMP2B	ETS1	HSPB1	MYH14	PSEN2	SQSTM1	VEGFA
APP	CLU	EWSR1	HSPB3	NEFH	PTK2B	SUSD2	VPS54
AR	COQ2	FBXO38	HSPB8	NHLRC1	RAB38	TAF15	WDR45
ASAH1	CR1	FERMT2	HTR7	NME8	REEP1	TARDBP	ZNF512B
ASCC1	CSF1R	FGGY	IGHMBP2	NOS3	REST	TBK1	TRPM7
ATP13A2	CTSC	FIG4	INPP5D	NOTCH3	SETX	TFG	SLC52A3
ATP7A	CYP27A1	FMNL1	ITM2B	NPC1	SIGMAR1	TMEM106B	PLAU
ATXN2	CYP2C19	FUS	ITPR2	OPTN	SLC1A1	TREM2	LRRK2
BICD2	DAO	GARS	KIF20B	PFN1	SLC1A2	TRIB3	GRB2
BIN1	DCTN1	GBA	LRP1	PICALM	SLC52A2	TRIP4	DNAJB2
BLMH							

### Genotype-Phenotype Correlation Analysis and Protein Interaction Network Construction

Patients with gene variants were divided into three groups according to initial symptoms, which included hypomnesia, mood problems, and behavioral changes. Genotype-phenotype was analyzed through a contingency table in GraphPad Prism software. To understand the protein–protein interaction (PPI), a PPI network was created using the Search Tool for Retrieval of Interaction Genes (STRING) database.

## Results

### Demographic and Clinical Variables

We identified five reported pathogenic/likely pathogenic variants in AD or FTD, one novel pathogenic variant, three novel likely pathogenic variants, and 21 variants with uncertain significance in AD or FTD (Tables 2, 3). No pathogenic expansions in *C9orf72* were detected in suspectable patients with FTD. Among all patients, 75% (63/84) patients developed hypomnesia as an initial symptom, 10.71% (9/84) developed mood problems, and 14.29% (12/84) developed behavioral changes. *APOE*ε4 allele was not more common in the variant group. *APOE*ε4 alleles occurred in 27.60% of patients in the gene mutation group and 50.90% of patients in the non-gene mutation group.

**TABLE 2:** Variants in cases with pathogenicity or uncertain significance.

**Case**	**APOE**	**Gene**	**Transcript No. (NM)**	**Mutation (CDS)**	**Change of amino acid**	**Age onset**	**Sex**	**Family history**	**Clinical diagnosis**	**Frequency prediction**				**Software prediction**			**ClinVar**	**ACMG**
										**dbSNP**	**HGMD**	**1000g**	**gnomAD v2.1.1**	**Plyphen2**	**Mutation taster**	**M-CAP**		
001	ε3/ε3	*PSEN1*	000021	A344G	Y115C	49	M	Yes	AD	Include	Include	NA	NA	Likely DC	DC	PP	P/LP	P
002	ε3/ε3	*APP*	000484	G2149A	V717I	56	M	Yes	AD	Include	Include	NA	NA	DC	DC	PP	P	P
003	ε3/ε3	*MAPT*	005910	G1165A	G389R	27	M	NA	FTD	Include	Include	NA	0.00005438	DC	DC	PP	LP	P
005	ε3/ε4	*SQSTM1*	003900	C671A	S224X	59	F	NA	FTD	NA	NA	NA	NA	/	DC	PP	NA	PATH
006	ε3/ε4	*PSEN2*	000447	T437C	I146T	59	F	Yes	AD/FTD	NA	NA	NA	0.00005437	DC	DC	PP	NA	VUS
007	ε3/ε3	*PSEN2*	000447	G100A	G34S	75	M	Yes	AD-PD	Include	NA	Include	0.006120	Benign	DC	PP	CIP	LP
008	ε3/ε3	*PSEN2*	000447	A785G	Y262C	81	M	Yes	AD^*^	NA	NA	NA	NA	DC	DC	PP	NA	VUS
009	ε3/ε3	*MAPT*	005910	G742A	V248M	57	M	NA	FTD**^#^**	Include	NA	Include	0.0001088	DC	DC	PP	P	P
010	ε2/ε4	*GRN*	002087	T617C	V206A	57	F	Yes	FTD**^#^**	NA	NA	NA	0.0002175	Likely DC	PM	PP	NA	VUS
011	ε3/ε3	*ERBB4*	005235	T2136G	I712M	56	F	NA	FTD-ALS	NA	NA	NA	NA	DC	DC	PP	NA	VUS
012	ε3/ε3	*C9orf72*	018325	T1442A	F481Y	63	M	NA	AD	NA	NA	NA	NA	DC	DC	PP	NA	VUS
013	ε2/ε3	*ABCA7*	019112	G2653C	V885L	44	F	NA	AD	Include	NA	NA	NA	Benign	PM	PP	NA	VUS
014	ε3/ε4	*ABCA7*	019112	C4690T	R1564X	62	F	Yes	AD	NA	NA	NA	0.00005438	DC	DC	PP	NA	LP
015	ε4/ε4	*ABCA7*	019112	T2933C	I978T	51	F	NA	AD/FTD	NA	NA	NA	0.0006522	DC	DC	PP	NA	VUS
016	ε3/ε3	*ADAM10*	001110	G502A	G168S	67	M	NA	AD/FTD	NA	NA	NA	NA	DC	DC	PP	NA	VUS
017	ε3/ε3	*ATP13A2*	001141973	A2281G	M761V	59	M	Yes	DLB	NA	NA	NA	NA	DC	DC	LB	NA	VUS
018	ε3/ε3	*ATP13A2*	022089	3135delC	Y1045X	52	F	NA	AD	NA	NA	NA	NA	DC	DC	/	NA	LP
019	ε3/ε4	*MPO*	000250	C1120T	R374W	63	M	Yes	AD^*^	NA	NA	NA	NA	DC	DC	PP	NA	VUS
020	ε2/ε3	*NOS3*	000603	2897-2A > G	/	68	F	NA	AD	NA	NA	NA	NA	DC	DC	/	NA	LP
021	ε3/ε3	*NOS3*	000603	1788dupT	S596fs	54	F	NA	AD^*^	Include	NA	Include	NA	/	DC	/	NA	VUS
022	ε3/ε3	*NOTCH3*	000435	G4240A	G1414S	53	M	NA	AD-NPH	NA	NA	NA	NA	DC	DC	PP	NA	VUS
024	ε3/ε4	*NOTCH3*	000435	G182T	R61L	64	F	NA	AD-VD	NA	NA	NA	NA	Benign	PM	PP	NA	VUS
026	ε3/3	*NOTCH3*	000435	C1715T	P572L	60	M	Yes	AD	NA	Include	NA	NA	DC	DC	PP	NA	VUS
027	ε3/ε3	*PTK2B*	004103	C1451T	P484L	65	M	Yes	AD-CAA	NA	NA	NA	0.0000544	DC	DC	PP	NA	VUS
028	ε3/ε3	*SETX*	015046	A3890G	Y1297C	69	F	NA	AD	NA	NA	NA	NA	/	PM	PP	NA	VUS
029	ε3/ε3	*SORL1*	003105	G1081C	V361L	28	F	NA	FTD**^#^**	NA	NA	NA	0.0003806	DC	DC	LB	NA	VUS
030	ε3/ε4	*SORL1*	003105	A296G	N99S	55	F	Yes	AD	NA	NA	NA	0.001033	DC	DC	LB	NA	VUS
031	ε3/ε3	*NEFH*	021076	C373G	L125V	62	F	Yes	AD	NA	NA	NA	0.0001449	DC	DC	PP	NA	VUS
032	ε3/ε4	*SYNJ1*	003895	T4664C	L1555P	72	M	NA	DLB	NA	NA	NA	NA	Benign	PM	LB	NA	VUS
		*SYNJ1*	003895	G317A	R106Q					NA	NA	NA	NA	Likely DC	DC	PP	VUS	VUS

^*^AD accurate diagnosis by Aβ biomarkers in CSF or PET.

*^#^Exclusion of AD diagnosis by Aβ biomarkers in CSF or PET.*

*CDS, coding sequence; Het, heterozygous; dbSNP, The Single Nucleotide Polymorphism Database; HGMD, Human Gene Mutation Database; 1000g, 1000 Genomics Projects; gnomAD, Genome Aggregation Database; M-CAP, Mendelian Clinically Applicable Pathogenicity; ACMG, American College of Medical Genetics and Genomics standards and guideline; DC, disease causing; PM, polymorphism; VUS, variant of uncertain significance; NA, not available; PP, possibly pathogenic; P, pathogenic; LP, likely pathogenic; CIP, conflicting interpretations of pathogenicity.*

**TABLE 3:**
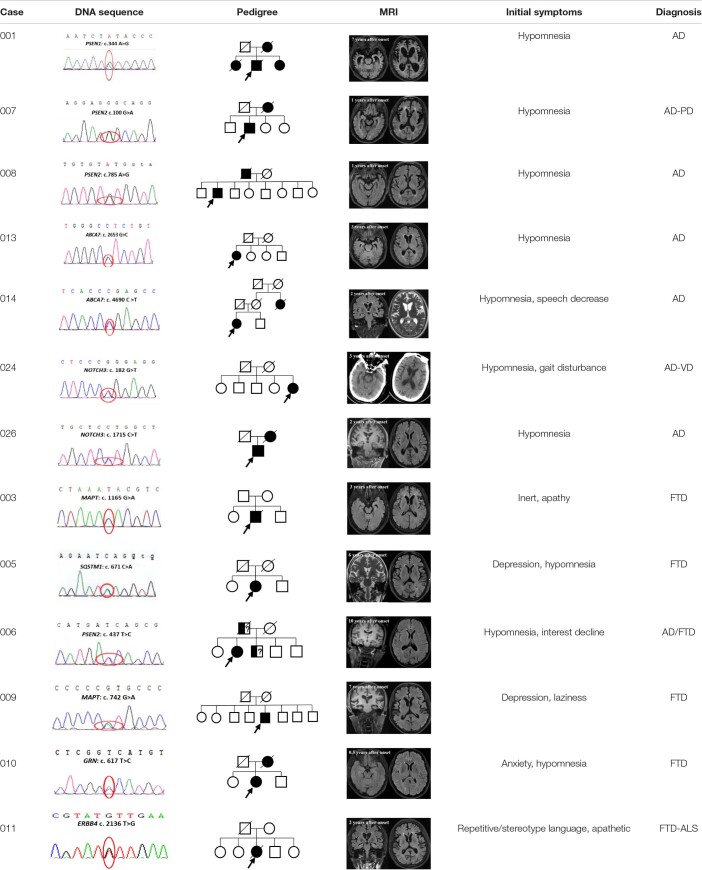
DNA sequence, brain MRI or CT, pedigree, initial symptoms, and clinical diagnosis of dementia patients with variants.

### Phenotypes of Patients With Alzheimer’s Disease and Associated Variants

**FIGURE 1:**
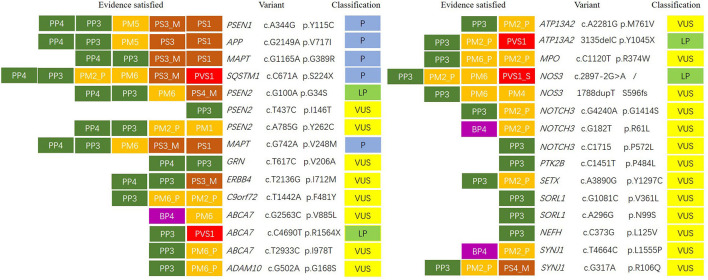
Variant classification according to American College of Medical Genetics and Genomics/Association for Molecular Pathology (ACMG/AMP) categorical rules. The first letter of each evidence indicated support toward a pathogenic (P) or benign (B) classification, and the second letter indicated the assigned evidence strength: very strong (VS), strong (S), moderate (M), or supporting (P). Evidence boxes were colored by evidence strength. Final classification was determined by the combinations of evidence resulting in the following: pathogenic (P), likely pathogenic (LP), or variant of uncertain significance (VUS).

We identified three known pathogenic/likely pathogenic variants in *PSEN1*, *PSEN2*, and *APP* in patients with AD, respectively. No. 001 presented with short memory disturbance at the age of 49. In the following 2 years, he could not work as normal. He got lost, became irritable, and showed visual hallucinations at the age of 54. He suffered from slow gait and suspicion at the age of 55, had to stay in bed at the age of 60, and died of severe pneumonia at the age of 62. Brain MRI revealed that brain atrophy and his MMSE and MoCA scores were both 0/30 with 16 years of education at the age of 58. His mother and two sisters had developed similar symptoms. The known *PSEN1* variant (NM_000021: c.A344G, p.Y115C) (Cruts et al., 1998) was found in this patient. No. 002 was admitted at the age of 56 with memory impairment. In the following years, his activities of daily living declined gradually. He was easy to become irritable, got lost, and had incontinence at the age of 62. His father also had the same symptoms at the age of 50+. The known *APP* variant (NM_000484: c.G2149A, p.V717I) (Jiao et al., 2014) was detected in this patient. No. 007 with *PSEN2* (NM_000447: c.G100A, p.G34S) (Jia et al., 2020) developed memory deficit at the age of 74 and got lost at the age of 75. He walked unsteadily, could not get benefits from the anti-Parkinson’s disease treatment at the age of 76, and had to stay in bed due to stiff limbs at the age of 78. Brain MRI showed multiple ischemic foci of the bilateral frontal-parietal lobe and brain atrophy (Tables 2, 3 and Figure 1).

We identified three novel likely pathogenic variants in *ABCA7*, *ATP13A2*, and *NOS3* in patients with AD. No. 014, carrying *ABCA7* variant (NM_019112: c.C4690T, p.R1564X), which produced a truncated protein with 583 amino acids less than normal protein, developed memory problem and speech deficit at the age of 62. Of note, 2 years after onset, her MMSE score was 16/30, and her MoCA was 14/30 with 9 years of education. Notably, 4 years later, she always wore one suit of clothes. At the age of 67, she often ignored her family members and could not look after herself. At the age of 68, she did not know her children but only her husband. Brain MRI revealed bilateral hippocampal atrophy, brain atrophy, and hyperintensity spots beside the left radiation coronal area. No. 018 developed hypomnesia at the age of 52 and was diagnosed with AD at the age of 54. Her MMSE score was 21/30 with 10 years of education, and brain MRI revealed mild demyelination of paraventricular white matter and brain atrophy at the age of 54. She was identified to harbor the variant *ATP13A2* (NM_022089: c.3135delC, p.Y1045X), which resulted in protein truncation with 136 amino acids less than the normal protein (Table 3). No. 020 became forgetful at the age of 68 and progressed to dementia at the age of 72. Her MMSE score was 20/30 with 12 years of education at the age of 72. She was identified to harbor *NOS3* (NM_000603: c.2897-2A > G), which existed at the junction of intron 23 and exon 24 and caused protein truncation (Tables 2, 3 and Figure 1).

We identified *PSEN2*, *C9orf72*, *NOTCH3*, and *NOS3* variants with uncertain significance in patients with AD and also detected six variants with uncertain significance in *ABCA7*, *MPO*, *SETX*, *SORL1*, and *NEFH*. No. 008 with *PSEN2* (NM_000447: c.A785G, p.Y262C) developed hypomnesia at the age of 81, got lost, and suffered from behavior disturbance after 6 months. His MMSE score was 23/30 and MoCA 18/30 with 16 years of education at the age of 65. On cerebrospinal fluid (CSF) examination, the level of Aβ (494.1 pg/ml) decreased and phosphorylated tau (p-tau; 110.23 pg/ml) increased. Brain MRI showed multiple cavity infarctions of bilateral radiation coronal area and frontal-parietal lobe, moderate leukoaraiosis, and brain atrophy. No. 012, carrying *C9orf72* variant (NM_018325: c.T1442A, p.F481Y), was admitted with memory disturbance at the age of 63. His MMSE score was 15/30 and MoCA 20/30 with 12 years of education at the age of 65. No. 021 presented with memory disturbance at the age of 54 and poor activities of daily living at the age of 59. On CSF examination, the level of Aβ decreased and p-tau increased. In the following 5 years, she suffered from eating difficulty and weight loss. She became receptive to aphasia and irritability. The *NOS3* variant (NM_001160110: c.1788dupT, p.S596fs), which resulted in the termination codon appearing ahead of schedule and a truncated protein with 18 amino acids less than the normal protein, was identified in this patient. No. 022, 024, and 026 were all identified to harbor the *NOTCH3* variants (NM_000435). No. 022 showed memory disturbance at the age of 53. He had difficulty in walking and dressing, as well as became urinary incontinent at the age of 61. His MoCA score was 1/30 with 6 years of education, and brain MRI revealed ischemic foci of bilateral basal ganglia, mild leukoaraiosis, and brain atrophy at the age of 63. Under CSF shunt operation, his function of walking and cognition were improved at the age of 64 but began to decline after 2 months. No. 024 presented with a memory problem at the age of 63 and an unsteady movement at the age of 65. She had difficulties in calculation at the age of 66, lost the way home, and became agnosia at the age of 70. Her MMSE and MoCA scores were both 0/30 with 6 years of education, and brain CT showed infarction of right basal ganglia, leukoaraiosis, and brain atrophy at the age of 66. No. 026 developed hypomnesia at the age of 60. He was in a delusion of being stolen, became grouchy, and often quarreled with others at the age of 63. His MoCA score was 6/30 with 12 years of education, and brain MRI revealed mild brain atrophy at the age of 62 (Tables 2, 3 and Figure 1).

### Phenotypes of Patients With Frontotemporal Dementia and Associated Variants

We identified two reported pathogenic variants in *MAPT*, one novel pathogenic variant in *SQSTM1*, and three variants with uncertain significance in *ERBB4*, *GRN*, and *SORL1* in patients with FTD. No. 003 with *MAPT* (NM_005910: c.G1165A, p.G389R) (Bermingham et al., 2008) was identified and reported by our team in 2017 (Sun et al., 2017). Then, he had to stay in bed at the age of 30 and died of severe pneumonia at the age of 33. No. 009 with *MAPT* variant (NM_005910: c.G742A, p.V248M) depressed and suspected himself seriously ill at the age of 56. In the following 2 years, he was apathetic and irritable. He was forgetful, stubborn, and ate only one type of food at the age of 60, and urinated anywhere at the age of 63. Brain MRI showed brain atrophy, especially in the bilateral anterior temporal lobe and hippocampus. No. 005 with *SQSTM1* variant (NM_003900: c.C671A, p.S224X) was identified and reported by our team in 2018 (Sun et al., 2018), which resulted in the premature termination of protein synthesis and a predicted truncated protein. However, we did not detect truncated proteins in this variant overexpressing HEK-293T cells because of the degradability of truncated protein. We identified the novel pathogenic *SQSTM1* S224X variant with loss of SQSTM1/p62 protein expression probably due to *SQSTM1* gene haploinsufficiency. Then, she exhibited hypertonia, unsteady gait, irritability, language dysfunction at the age of 67 and showed visual hallucination and dysphagia at the age of 68. No. 011 with *ERBB4* (NM_005235: c.T2136G, p.I712M) was identified and reported by our team in 2020 (Sun et al., 2020). Through follow-up, we learned that this patient died at home at the age of 62. No. 010 felt depressed, anxious, and forgetful at the age of 57. In the following 3 years, her condition gradually aggravated, and her abilities to work were influenced. Her MoCA score was 14/30 with 14 years of education. *GRN* variant (NM_002087: c.T617C, p.V206A) was identified in this patient (Tables 2, 3 and Figure 1).

### Phenotypes of Patients With Uncertainty Whether Possible Alzheimer’s Disease or Frontotemporal Dementia and Associated Variants

We identified three variants with uncertain significance in *PSEN2*, *ABCA7*, and *ADCM10*. No. 006 showed memory impairment at the age of 59, felt depressed and interest declined at the age of 61 and acted in a strange way, such as putting the jacket down in the close stool at the age of 64. In the following 7 years, she was suspicious and became urinary incontinent. The *PSEN2* (NM_000447: c.T437C, p.I146T) was identified in this patient. No. 015 with *ABCA7* (NM_019112: c.T2933C, p.I978T) became irritable at the age of 49, often forgot recent things, and suffered from poor ability in writing at the age of 51. She could not take care of herself and lose weight at the age of 55. Her MMSE and MoCA were all 0/30 with 12 years of education at the age of 55 (Tables 2, 3 and Figure 1).

### Genotype-Phenotype Correlation and Protein–Protein Interaction Network

**FIGURE 2:**
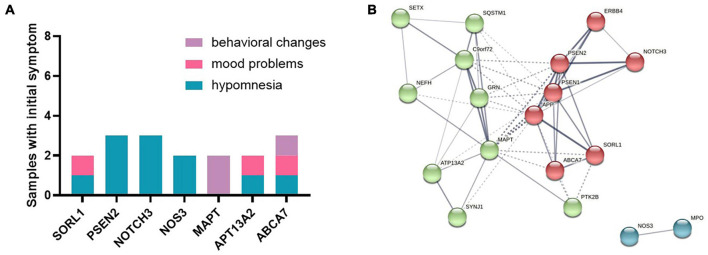
**(A)** Initial symptoms of patients with variants in *SORl1*, *PSEN2*, *NOTCH3*, *NOS3*, *MAPT*, *APT13A2*, and *ABCA7* genes (samples *n* ≥ 2). **(B)** The protein–protein interaction (PPI) network showed that there were three clusters among all proteins based on the K-means clustering algorithm.

Initial symptoms of patients were with variants in *SORl1*, *PSEN2*, *NOTCH3*, *NOS3*, *MAPT*, *APT13A2*, and *ABCA7* genes (samples *n* ≥ 2) (Figure 2A). Hypomnesia was the most common initial symptom among all patients, behavioral change was inclined to occur in *MAPT* and *ABCA7* variant carriers, and mood problems usually happened to the subjects with *SORL1*, *APT13A2*, and *ABCA7* variants. PPI networks showed that there were three clusters based on the K-means clustering algorithm among all proteins (Figure 2B). NOS3 and MPO proteins had no relationships with other proteins. Several important proteins such as APP, PSEN1, PSEN2, MAPT, C9orf72, and GRN, *etc*., had more interactions with other proteins.

## Discussion

This study aimed to find genetic variants contributing to clinical phenotypes of degenerative dementias. First, we identified five reported pathogenic/likely pathogenic variants in patients with AD or FTD. Second, we identified 1 novel pathogenic variant in *SQSTM1* genes, 3 novel likely pathogenic variants in *ABCA7*, *ATP13A2*, and *NOS3*, and 21 variants with uncertain significance in *PSEN2*, *C9orf72*, *NOTCH3*, *ABCA7*, *ERBB4*, *GRN*, *MPO*, *SETX*, *SORL1*, *NEFH*, *ADCM10*, and *SORL1*, *etc.*, in patients with AD or FTD.

The clinical symptoms of the *PSEN1* Y115C variant were rarely reported in the previous literature. Our case, with 13 years of the disease duration, was featured with early memory disturbance, visuospatial disorientation, behavioral and psychological symptoms, dysphasia, and motor disorder. The mean onset age of this variant reported was 42 years (range 39–49 years) (van Duijn et al., 1994), which was similar to our case (49 years). There was the observation of later occurrence of symptoms in variants after codon 200 in *PSEN*1 compared with those before it in EOAD (Ryan and Rossor, 2010). Furthermore, it was concluded that the patient with *PSEN1* missense variants located in hydrophobic regions had an earlier age onset than those with variants located in non-hydrophobic regions, and *PSEN1* Y115C just existed in the hydrophobic region. This phenomenon was that variants in hydrophobic regions affected the γ-secretase active site, promoting the aggregation of Aβ, and eventually inducing the onset of AD (Jia et al., 2020).

*APP* V717I was identified in a previous pedigree (Murrell et al., 2000), which showed the clinical onset of disease in their mid-to-late 30s, with short-term memory deficit, gradually worsening the problems of visuospatial, executive, and expressive language functions. The disease duration is approximately 10 years based on the data of the family history. The pathological feature of this variant is the high production of Aβ fibril deposits (Murrell et al., 1991). No. 002 developed memory disturbance at the age of 56 and progressed that he got lost and incontinence in 6 years. His father also had the same symptoms at the age of 50+. Our investigations broadened the phenotype of AD with *APP* variants.

*PSEN2* G34S variant was reported to relate to AD and mild cognitive impairment (MCI). This variant was identified in three pedigrees with the older mean age of onset (69, 52, 75, respectively) (Jia et al., 2020), which suggested relatively weak pathogenicity. No. 007 with *PSEN2* G34S developed memory disturbance at the age of 74 but progressed fast that he had to stay in bed in 4 years. Although there were fewer variants with late age onset in familial AD than variants with early age onset, they certainly existed. Furthermore, we explored two variants with uncertain significance in *PSEN2*, which may contribute to the disease occurrence. No. 006, carrying *PSEN2* T437C variant, developed memory disturbance at the age of 59 and showed an obvious change of personality and behavior in the next 5 years. The patient has identified the clinical diagnosis with uncertainty whether possible AD or FTD. No. 008, with *PSEN2* A785G variant, developed memory deficit at the age of 81, and the CSF examination identified the diagnosis of AD. These two variants were predicted to be damaging by three predictive algorithms, indicating the pathogenicity of these variants.

In this study, five missense variants were first identified to be related to FTD. Among these variants, two (i.e., *SQSTM1* C671A and *ERBB4* T2136G) were first reported by our team (Sun et al., 2018, 2020) and were confirmed function deficits of the target proteins. *MAPT* G389A was previously reported to affect a 17-year-old girl with the initial symptoms of atypical depression and emotional blunting and a 21-year-old woman with the initial symptom of postpartum depression. No. 003 also presented with the cognitive disorder at the age of 27 (Sun et al., 2017). It seemed that the early-onset cases of FTD were more likely to be found in tau G389R carriers. Tau G389R variant in the pseudo-repeat region (PRR) could increase microtubule dynamicity (Niewidok et al., 2016), and V248M variant impaired homeostatic control of spontaneous neuronal activity in response to depolarization (Sohn et al., 2019). Through our analysis, behavioral problem as the initial symptom was inclined to occur in *MAPT* variant carriers.

We explored three variants in *NOTCH3* with uncertain significance, which might contribute to the occurrence of AD. This gene encoding a single-pass transmembrane protein, predominantly expressed in vascular smooth muscle cells, and got involved in the mechanism of cerebral autosomal dominant arteriopathy with subcortical infarcts and leukoencephalopathy (CADASIL) (Mašek and Andersson, 2017). Among these three missense variants, two were predicted to be damaging by at least two predictive algorithms (i.e., G4240A and G182T), and one was predicted as a polymorphism but did not find in dbSNP, HGMD, 1000g, and gnomAD database. A hypothesis of AD suggested that, in CADASIL, triggering events in the pathogenic cascade were not amyloid deposits but damaged blood vessels caused by inflammatory reactions that led to ischemia, amyloid accumulation, and axonal degeneration (Bonvicini et al., 2019). Variants in the *NOTCH3* gene were known to provoke inflammatory reactions (Marchesi, 2016). Furthermore, we explored the one variant in *NOS3* with likely pathogenicity in patients with AD. Nitric oxide performed vital physiological functions in the nervous and other systems under normal concentrations, but persistently high levels could create a toxic environment. *NOS3* was a strong candidate susceptibility gene for Parkinsonism syndromes (Hancock et al., 2008). Some studies genotyped EOAD and late-onset AD (LOAD) for *NOS3* polymorphism, which found the associate of *NOS3* gene with LOAD. This effect that is independent of *APOE* genotype concluded that *NOS3* may be a new genetic risk factor for LOAD (Styczyńska et al., 2008; Wang et al., 2008; Azizi et al., 2010). Our analysis also showed that NOS3 had no interaction with other proteins except MPO, which suggested that the pathway of the *NOS3* gene was different from other dementia-related genes. PPI network that demonstrated proteins such as PSEN1, PSEN2, APP, MAPT, and C9orf72 had abundant interactions with other proteins, which was consistent with the important roles of these dementia pathogenic genes.

Furthermore, our study reported that the *APOE*ε4 allele was not more common in the gene variant group. Of note, 27.60% of patients in the gene variant group and 50.90% of patients in the non-gene mutation group presented with one or two *APOE*ε4 alleles. Thus, it was possible that *APOE*ε4 did not act as a promoter of the clustering of AD with gene variants. Other unidentified genetic variants or environmental factors, acting independently or in concert, were involved in the development of dementia (Jia et al., 2020).

Our study had several limitations. The progression history of the disease was collected based on the recall of relatives with normal cognition, but still, there was a recall inaccuracy. In most cases of patients with AD, neither amyloid PET nor CSF Aβ biomarkers were included to corroborate the diagnosis. There was a limited ability to assess causality when screening individuals without affected and unaffected family members. The pathogenicity of variants with uncertain significance should be verified by large-scale studies and functional experiments. All patients involved in our analysis were severe, but the severity of patients was not strictly correlated with the findings of the pathogenic variants. Due to its incapability of detecting large segment INDEL, there were still many patients without detectable variants through exome sequencing, which could fail to explore the genetic factors in a subset of these patients with dementia. Genetic screening could not determine all the causes of dementia.

## Conclusion

The WES techniques had the potential to give a genetic diagnosis for some intractable dementia associated with the heterogeneity of clinical manifestations. This study also expanded the clinical spectrums of AD and FTD. The pathogenicity of the mentioned known genetic variants had been most appreciated in the European population, and this study showed the novelty of genetic variations in degenerative dementia in the Asian population.

## Data Availability Statement

According to the national legislation/guidelines, specifically the Administrative Regulations of the People’s Republic of China on Human Genetic Resources (http://www.gov.cn/zhengce/content/2019-06/10/content_5398829.htm, http://english.www.gov.cn/policies/latest_releases/2019/06/10/content_281476708945462.htm), no additional raw data are available at this time. Data of this project can be accessed after an approval application to the China National Genebank (CNGB, https://db.cngb.org/cnsa/). Please refer to https://db.cngb.org/, or email: CNGBdb@cngb.org for detailed application guidance. The accession code CNP0002218 should be included in the application.

## Ethics Statement

The studies involving human participants were reviewed and approved by the Shanghai Mental Health Center Ethical Standards Committee on human experimentation. The patients/participants provided their written informed consent to participate in this study. Written informed consent was obtained from the individual(s) for the publication of any potentially identifiable images or data included in this article.

## Author Contributions

LS analyzed the data, wrote and revised the manuscript. NS and JZ collected the clinical data of patients. SZ, FY, and XinL assessed the cognitive function of patients. WL analyzed the gene data. SX and XiaL designed and supervised the experiment. All authors contributed to the article and approved the submitted version.

## Conflict of Interest

The authors declare that the research was conducted in the absence of any commercial or financial relationships that could be construed as a potential conflict of interest.

## Publisher’s Note

All claims expressed in this article are solely those of the authors and do not necessarily represent those of their affiliated organizations, or those of the publisher, the editors and the reviewers. Any product that may be evaluated in this article, or claim that may be made by its manufacturer, is not guaranteed or endorsed by the publisher.

## References

[B1] Azizi Z. Noroozian M. Kaini-Moghaddam Z. Majlessi N. (2010). Association between NOS3 gene G894T polymorphism and late-onset Alzheimer disease in a sample from Iran. *Alzheimer Dis. Assoc. Disord.* 24 204–208. 10.1097/wad.0b013e3181a7c8fd 20505439

[B2] Bermingham N. Cowie T. F. Paine M. Storey E. McLean C. (2008). Frontotemporal dementia and Parkinsonism linked to chromosome 17 in a young Australian patient with the G389R Tau mutation. *Neuropathol. Appl. Neurobiol.* 34 366–370. 10.1111/j.1365-2990.2007.00918.x 18067537

[B3] Bettens K. Sleegers K. Van Broeckhoven C. (2013). Genetic insights in Alzheimer’s disease. *Lancet. Neurol.* 12 92–104. 23237904 10.1016/S1474-4422(12)70259-4

[B4] Bonvicini C. Scassellati C. Benussi L. Di Maria E. Maj C. Ciani M. (2019). Next generation sequencing analysis in early onset dementia patients. *J. Alzheimers Dis.* 67 243–256. 30530974 10.3233/JAD-180482 PMC6398561

[B5] Chaudhury S. Patel T. Barber I. S. Guetta-Baranes T. Brookes K. J. Chappell S. (2018). Polygenic risk score in postmortem diagnosed sporadic early-onset Alzheimer’s disease. *Neurobiol. Aging* 62 244.e1–244.e8. 10.1016/j.neurobiolaging.2017.09.035 PMC5995122 29103623

[B6] Cruts M. van Duijn C. M. Backhovens H. Van den Broeck M. Wehnert A. Serneels S. (1998). Estimation of the genetic contribution of presenilin-1 and -2 mutations in a population-based study of presenile Alzheimer disease. *Hum. Mol. Genet.* 7 43–51. 10.1093/hmg/7.1.43 9384602

[B7] Gorno-Tempini M. Hillis A. E. Weintraub S. Kertesz A. Mendez M. Cappa S. F. (2011). Classification of primary progressive aphasia and its variants. *Neurology* 76 1006–1014. 21325651 10.1212/WNL.0b013e31821103e6 PMC3059138

[B8] Hancock D. Martin E. R. Vance J. M. Scott W. K. (2008). Nitric oxide synthase genes and their interactions with environmental factors in Parkinson’s disease. *Neurogenetics* 9 249–262. 10.1007/s10048-008-0137-1 18663495 PMC2630458

[B9] Jia L. Fu Y. Shen L. Zhang H. Zhu M. Qiu Q. (2020). PSEN1, PSEN2, and APP mutations in 404 Chinese pedigrees with familial Alzheimer’s disease. *Alzheimers Dement.* 16 178–191. 10.1002/alz.12005 31914229

[B10] Jiao B. Tang B. Liu X. Xu J. Wang Y. Zhou L. (2014). Mutational analysis in early-onset familial Alzheimer’s disease in Mainland China. *Neurobiol. Aging* 35 1957.e1–1957.e6. 10.1016/j.neurobiolaging.2014.02.014 24650794

[B11] Koriath C. Kenny J. Ryan N. S. Rohrer J. D. Schott J. M. Houlden H. (2020). Genetic testing in dementia – utility and clinical strategies. *Nat. Rev. Neurol.* 17 23–36. 10.1038/s41582-020-00416-1 33168964

[B12] Li M. Datto M. Duncavage E. J. Kulkarni S. Lindeman N. I. Roy S. (2017). Standards and guidelines for the interpretation and reporting of sequence variants in cancer: a joint consensus recommendation of the association for molecular pathology, American society of clinical oncology, and college of American pathologists. *J. Mol. Diagn.* 19 4–23. 27993330 10.1016/j.jmoldx.2016.10.002 PMC5707196

[B13] Marchesi V. (2016). Gain-of-function somatic mutations contribute to inflammation and blood vessel damage that lead to Alzheimer dementia: a hypothesis. *FASEB J.* 30 503–506. 10.1096/fj.15-282285 26527064

[B14] Mašek J. Andersson E. (2017). The developmental biology of genetic Notch disorders. *Development (Camb. Engl.)* 144 1743–1763. 10.1242/dev.148007 28512196

[B15] McKhann G. Knopman D. S. Chertkow H. Hyman B. T. Jack C. R. Jr. Kawas C. H. (2011). The diagnosis of dementia due to Alzheimer’s disease: recommendations from the National institute on aging-Alzheimer’s association workgroups on diagnostic guidelines for Alzheimer’s disease. *Alzheimers Dement.* 7 263–269. 21514250 10.1016/j.jalz.2011.03.005 PMC3312024

[B16] Murrell J. Farlow M. Ghetti B. Benson M. D. (1991). A mutation in the amyloid precursor protein associated with hereditary Alzheimer’s disease. *Science (New York, N.Y.).* 254 97–99. 10.1126/science.1925564 1925564

[B17] Murrell J. Hake A. M. Quaid K. A. Farlow M. R. Ghetti B. (2000). Early-onset Alzheimer disease caused by a new mutation (V717L) in the amyloid precursor protein gene. *Arch. Neurol.* 57 885–887. 10.1001/archneur.57.6.885 10867787

[B18] Nicolas G. Veltman J. (2019). The role of de novo mutations in adult-onset neurodegenerative disorders. *Acta Neuropathol.* 137 183–207. 10.1007/s00401-018-1939-3 30478624 PMC6513904

[B19] Niewidok B. Igaev M. Sündermann F. Janning D. Bakota L. Brandt R. (2016). Presence of a carboxy-terminal pseudorepeat and disease-like pseudohyperphosphorylation critically influence tau’s interaction with microtubules in axon-like processes. *Mol. Biol. Cell* 27 3537–3549. 10.1091/mbc.e16-06-0402 27582388 PMC5221586

[B20] Nygaard H. Erson-Omay E. Z. Wu X. Kent B. A. Bernales C. Q. Evans D. M. (2019). Whole-Exome sequencing of an exceptional longevity cohort. *J. Gerontol. A Biol. Sci. Med. Sci.* 74 1386–1390. 10.1093/gerona/gly098 29750252 PMC6696723

[B21] Olszewska D. Lonergan R. Fallon E. M. Lynch T. (2016). Genetics of frontotemporal dementia. *Curr. Neurol. Neurosci. Rep.* 16:107. 27878525 10.1007/s11910-016-0707-9

[B22] Rascovsky K. Hodges J. R. Knopman D. Mendez M. F. Kramer J. H. Neuhaus J. (2011). Sensitivity of revised diagnostic criteria for the behavioural variant of frontotemporal dementia. *Brain* 134 2456–2477. 21810890 10.1093/brain/awr179 PMC3170532

[B23] Ryan N. Rossor M. (2010). Correlating familial Alzheimer’s disease gene mutations with clinical phenotype. *Biomark. Med.* 4 99–112. 20387306 10.2217/bmm.09.92 PMC3937872

[B24] Seo J. Byun M. S. Yi D. Lee J. H. Jeon S. Y. Shin S. A. (2020). Genetic associations of in vivo pathology influence Alzheimer’s disease susceptibility. *Alzheimers Res. Ther.* 12:156. 33213512 10.1186/s13195-020-00722-2 PMC7678113

[B25] Sohn P. Huang C. T. Yan R. Fan L. Tracy T. E. Camargo C. M. (2019). Pathogenic Tau impairs axon initial segment plasticity and excitability homeostasis. *Neuron* 104 458–470.e5. 31542321 10.1016/j.neuron.2019.08.008 PMC6880876

[B26] Styczyńska M. Strosznajder J. B. Religa D. Chodakowska-Zebrowska M. Pfeffer A. Gabryelewicz T. (2008). Association between genetic and environmental factors and the risk of Alzheimer’s disease. *Folia Neuropathol.* 46 249–254. 19169966

[B27] Sun L. Chen K. Li X. Xiao S. (2017). Rapidly progressive frontotemporal dementia associated with MAPT mutation G389R. *J. Alzheimers Dis.* 55 777–785. 10.3233/jad-160802 27802239

[B28] Sun L. Cheng B. Zhou Y. Fan Y. Li W. Qiu Q. (2020). ErbB4 mutation that decreased NRG1-ErbB4 signaling involved in the pathogenesis of amyotrophic lateral sclerosis/frontotemporal dementia. *J. Alzheimers Dis.* 74 535–544. 10.3233/jad-191230 32065797

[B29] Sun L. Rong Z. Li W. Zheng H. Xiao S. Li X. (2018). Identification of a novel hemizygous SQSTM1 nonsense mutation in atypical behavioral variant frontotemporal dementia. *Front. Aging Neurosci.* 10:26. 10.3389/fnagi.2018.00026 29467647 PMC5808128

[B30] van Duijn C. Hendriks L. Farrer L. A. Backhovens H. Cruts M. Wehnert A. (1994). A population-based study of familial Alzheimer disease: linkage to chromosomes 14, 19, and 21. *Am. J. Hum. Genet.* 55 714–727. 7942850 PMC1918296

[B31] Wang B. Tan S. Yang Z. Xie Y. C. Wang J. Zhou S. (2008). Association between Alzheimer’s disease and the NOS3 gene Glu298Asp polymorphism in Chinese. *J. Mol. Neurosci.* 34 173–176. 10.1007/s12031-007-9026-6 18183499

[B32] Xu Y. Liu X. Shen J. Tian W. Fang R. Li B. (2018). The whole exome sequencing clarifies the genotype- phenotype correlations in patients with early-onset dementia. *Aging Dis.* 9 696–705. 10.14336/ad.2018.0208 30090657 PMC6065298

